# Detection of ZrO_2_ Nanoparticles in Lung Tissue Sections by Time-of-Flight Secondary Ion Mass Spectrometry and Ion Beam Microscopy

**DOI:** 10.3390/nano8010044

**Published:** 2018-01-15

**Authors:** Lothar Veith, Julia Böttner, Antje Vennemann, Daniel Breitenstein, Carsten Engelhard, Jan Meijer, Irina Estrela-Lopis, Martin Wiemann, Birgit Hagenhoff

**Affiliations:** 1 Tascon GmbH, Mendelstraße 17, 48149 Münster, Germany; daniel.breitenstein@tascon-gmbh.de (D.B.); birgit.hagenhoff@tascon-gmbh.de (B.H.); 2 Department of Chemistry & Biology, University of Siegen, Adolf-Reichwein-Straße 2, 57076 Siegen, Germany; engelhard@chemie.uni-siegen.de; 3 Institute for Medical Physics & Biophysics, Leipzig University, Härtelstraße 16-18, 04107 Leipzig, Germany; julia.boettner@medizin.uni-leipzig.de (J.B.); irina.estrela-lopis@medizin.uni-leipzig.de (I.E.-L.); 4 IBE R&D Institute for Lung Health gGmbH, Mendelstraße 11, 48149 Münster, Germany; vennemann@ibe-ms.de; 5 Felix-Bloch Institute for Solid State Physics, Leipzig University, Linnéstraße 5, 04103 Leipzig, Germany; jan.meijer@uni-leipzig.de

**Keywords:** ToF-SIMS, ion beam microscopy, particle-induced X-ray emission, ZrO_2_ nanoparticles, lung tissue, instillation, toxicity, gas cluster ion beam, delayed extraction

## Abstract

The increasing use of nanoparticles (NP) in commercial products requires elaborated techniques to detect NP in the tissue of exposed organisms. However, due to the low amount of material, the detection and exact localization of NP within tissue sections is demanding. In this respect, Time-of-Flight Secondary Ion Mass Spectrometry (ToF-SIMS) and Ion Beam Microscopy (IBM) are promising techniques, because they both offer sub-micron lateral resolutions along with high sensitivities. Here, we compare the performance of the non-material-consumptive IBM and material-consumptive ToF-SIMS for the detection of ZrO_2_ NP (primary size 9–10 nm) in rat lung tissue. Unfixed or methanol-fixed air-dried cryo-sections were subjected to IBM using proton beam scanning or to three-dimensional ToF-SIMS (3D ToF-SIMS) using either oxygen or argon gas cluster ion beams for complete sample sputtering. Some sample sites were analyzed first by IBM and subsequently by 3D ToF-SIMS, to compare results from exactly the same site. Both techniques revealed that ZrO_2_ NP particles occurred mostly agglomerated in phagocytic cells with only small quantities being associated to the lung epithelium, with Zr, S, and P colocalized within the same biological structures. However, while IBM provided quantitative information on element distribution, 3D ToF-SIMS delivered a higher lateral resolution and a lower limit of detection under these conditions. We, therefore, conclude that 3D ToF-SIMS, although not yet a quantitative technique, is a highly valuable tool for the detection of NP in biological tissue.

## 1. Introduction

The detection of nanoparticles (NP) in tissue sections is an important prerequisite to determine potential risks for human health upon nanoparticle exposition. Due to their small sizes and low quantities the unambiguous detection and localization of NP is a demanding task. Time-of-Flight Secondary Ion Mass Spectrometry (ToF-SIMS) shows a high potential to detect a great variety of NP composed of inorganic materials along with the organic and inorganic composition of tissue thin sections. However, due to small masses of NP signal intensities are low and an unambiguous identification is challenging. Low signal-to-noise ratios complicate the recognition of isotopic patterns of the nanomaterial’s elements. Furthermore, the size of a NP is smaller than a single pixel in the imaging analyses. Therefore, a second high resolution technique validating the identification and signal distribution of the ToF-SIMS results is desirable to prove the correct interpretation of the ToF-SIMS results at this stage of ToF-SIMS method development.

With a lateral resolution of the applied ToF-SIMS method of approximately 600 nm, we were able to identify fluorescent SiO_2_ NP in lung tissue sections [1], and this localization was validated by fluorescence microscopy. However, for the vast majority of NP not carrying a fluorescent label or not showing plasmonic resonance, other high resolution techniques need to be explored if one aims to validate ToF-SIMS results. In this respect, Ion Beam Microscopy (IBM) appears to be a well-suited technology, because its lateral resolution of ca. 1 µm is similar to the one of ToF-SIMS. The IBM technique combines the analysis of the particle-induced X-ray emission (PIXE) and the analysis of the energy of the backscattered particles, referred to as Rutherford backscattering spectrometry (RBS). This setup provides a high lateral resolution, undoubted element identification and their quantification [2,3,4]. As the technique is primarily non-destructive, successive analyses of tissue samples with IBM and ToF-SIMS should allow the detection of NP along with components of the biological matrix. ToF-SIMS detects molecules with a theoretically unlimited mass range. It allows the detection of organic as well as inorganic species, whereby molecular fragments are gathered from the surface which can be sputtered to obtain signals from deeper layers. IBM provides laterally resolved information on all element concentrations which, in contrast to ToF-SIMS, are always gathered from the whole sample depth. Thus, besides the benchmarking of the nanoparticle distribution complementary quantitative information should be accessible.

The aim of this study was to compare the results of ToF-SIMS and IBM analyses side-by-side using a 9–10 nm sized zirconium oxide (ZrO_2_) NP distributed in lung tissue. Several reasons prompted us to choose this particular preparation: Firstly, own pilot experiments have shown that ZrO_2_ NP gives rise to high signal intensities with both techniques, which is an important prerequisite for a comparative study. Secondly, ToF-SIMS and IBM should be compared with respect to their ability to detect small ZrO_2_ NP in tissue, and to derive further information on the distribution of tissue components, such as the minor and trace elements by IBM, and elements and organic fragments by ToF-SIMS. Ideally, comparative analyses should be performed at exactly the same spot of the sample. Thirdly, the study, which is part of the special issue on “Nanoparticles in Vivo and in Vitro Studies: a Collection of Parallel Approaches”, shall also contribute to our understanding of the toxicological relevance of ZrO_2_ NP in the lung, which acts as the main entry port for airborne nanomaterials into the body. ZrO_2_ NP are used in coatings of self-cleaning stoves, in dental filler material, or implant materials [5], and the toxic effects of differentially coated ZrO_2_ NP on alveolar macrophages and on rat and allergic mouse lung have been described in an accompanying publication [6]. Of note, ZrO_2_ NP administered to the lung has been previously located mainly within alveolar macrophages by Raman microspectroscopy, hyperspectral microscopy, and immunocytochemistry [6,7]. The present study comparing IBM and ToF-SIMS analyses of ZrO_2_ NP distribution was carried out on rat lung tissue from this previous study [6].

## 2. Results and Discussion

The first part (2.1) will describe the detection of zirconium (Zr), phosphorous (P), and sulfur (S) within a ZrO_2_ NP laden lung by IBM. Similarly, the subsequent parts (2.2 and 2.3) will describe a ToF-SIMS analysis together with inorganic and organic components. The last chapter (2.4) is devoted to the comparison of both techniques applied consecutively to the same position of a ZrO_2_ containing tissue section. Representative examples showing the microscopic structure of cryo-sections as used here are shown in an accompanying paper of this special issues [7].

### 2.1. Typical IBM Results for an Individual Position

IBM analysis typically consists of two components. Particle-induced X-ray emission (PIXE) yields a signal distribution of elements by analyzing the X-ray emission upon proton bombardment. The Rutherford backscattering spectrometry (RBS) analyzes the energy of backscattered protons and provides information on the elemental composition, depth profile, the accumulated charge, and the thickness of a sample.

**Figure 1:**
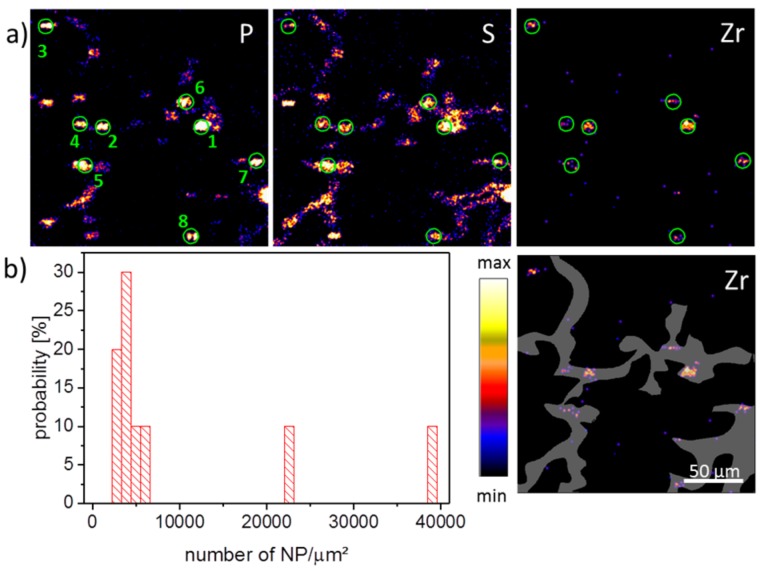
Particle-induced X-ray emission (PIXE) images of an air-dried lung section three days post instillation of ZrO_2_ nanoparticles. (**a**) Signal distributions for P, S, and Zr, and Zr with superimposed region-of-interest (ROI, grey area). P and S are assumed to be cellular components found within the tissue at elevated levels. The Zr signals resemble accumulations of nanoparticles; (**b**) Histogram of nanoparticle (NP) loading across a population of cells indicated by the green ROIs 1–8.

PIXE images of a ZrO_2_ containing tissue section three days post-instillation are shown in Figure 1a where the signal distribution of phosphorus, sulfur, and zirconium is presented. The grey area indicates a region-of-interest (ROI) comprising the network of alveolar septa, which are main constituents of the lung parenchyma. The phosphorus image showed numerous elliptical structures of high intensity, with diameters up to 20 µm. While frequency, distribution, and size of the smaller structures most likely represented phosphate groups of the nuclear DNA within cell nuclei, larger spots may have been caused by type-2 pneumocytes, which contain phospholipid-rich lamellar bodies as lung surfactant precursors, or alveolar macrophages, which also may engulf portions of lung surfactant. Furthermore, it had to be taken into account that the K line from phosphorus and L line from zirconium coincided with each other. Therefore the phosphor distribution was partially influenced by the Lα emission from the Zr signal. The sulfur distribution showed an overall medium intensity in alveolar septa with higher intensity areas in P-rich (nuclear) regions. The sulfur distribution reflected sulfur-containing amino acids in proteins, and the highest intensity should be found where the local protein concentration is high. Again, nuclear structures bearing DNA-histone complexes may underlie this phenomenon. The zirconium distribution showed some intense areas all of which are associated with P- and S-rich regions. This, and the fact that the diameters of these patches were mostly below 10 µm, suggested a significant concentration of nanoparticles and/or small agglomerates in single cells, like alveolar macrophages and other cells of the alveolar septa. The concentration of NPs in different patches detected in various PIXE images was calculated. The PIXE and RBS spectra were extracted from the eight ROIs which mark the NP spots by the green circles (Figure 1a). After fitting the spectra, the obtained concentration of Zr elements was recalculated in terms of NP number per µm^2^ assuming a density of ZrO_2_ of 5680 kg/m³ per NP with a mean size of 9 nm. Figure 1b shows the histogram of the NP loading across an evaluated lung section. The ROIs revealing a low NP loading of about 4000 NPs/µm^2^ were associated with the alveolar septa, composed of type-1 and type-2 pneumocytes. A higher NP loading of up to 40,000 NPs/µm^2^ was found in ROIs 1 and 2 (Figure 1b). It may be suggested that the high loading of NPs occurs in alveolar macrophages, which were in the close vicinity of alveolar septum.

### 2.2. Individual Position SIMS Analysis: Inorganic Depth Profiling

In the first step, three-dimensional ToF-SIMS analyses (3D ToF-SIMS) were carried out using O_2_^+^ sputtering, as this lowers the detection limit for the nanoparticles compared to Ar-cluster sputtering. O_2_^+^ sputtering ensures a high oxidation state of the Zr in the samples as it provides excess oxygen and fosters the secondary ion yield of the electropositive Zr for 3D ToF-SIMS analyses. Furthermore, due to the availability of O_2_ during the analysis, the maximum ionization yield of the Zr specific ZrO^+^ signal should be reached, which optimizes the signal-to-noise ratio for this channel.

**Figure 2:**
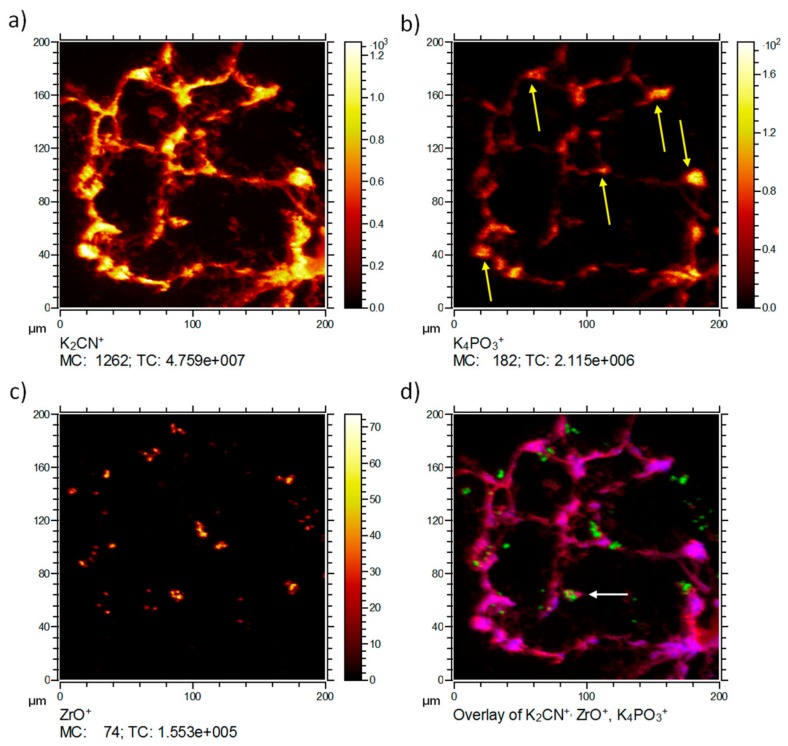
Time-of-Flight Secondary Ion Mass Spectrometry (ToF-SIMS) three-dimensional (3D) analysis of an air-dried lung section three days post instillation of ZrO_2_ nanoparticles. Lateral distributions of three secondary ions K_2_CN^+^, K_4_PO_3_^+^, and ZrO^+^ are shown upon O_2_^+^ sputtering. (**a**) K_2_CN^+^ shows the typical alveolar structure of a lung section; (**b**) K_4_PO_3_^+^ is found as patches within the tissue and with high intensity in alveolar edges (yellow arrows); (**c**) ZrO^+^ is concentrated in single patches attached to alveolar walls; the accumulations within the alveolar space most likely represent alveolar macrophages (white arrow); (**d**) A correlation analysis of all ions with K_2_CN^+^ in red, ZrO^+^ in green, and K_4_PO_3_^+^ in blue reveals the respective lateral signal distribution. The white arrow points to a macrophage-like cell. (MC: maximum counts per pixel, TC: total counts for the image).

ToF-SIMS analysis of a ZrO_2_ NP-containing lung tissue section three days post NP administration is presented in Figure 2. The 3D data set was projected onto a 2D plane to enable a simple comparison of signal distributions. Typically, the K_2_CN^+^ signal (*m*/*z* = 103.95, Figure 2a) is encountered in tissue material and used as a marker ion, if ToF-SIMS analysis of biomaterials is conducted with O_2_^+^ sputtering. The K_4_PO_3_^+^ signal (*m*/*z* = 234.91, Figure 2b) can be used as an indication for the phosphate distribution in K-rich tissue samples. In lung cells, this signal is therefore, mostly but not exclusively indicative of nuclear structures, and often surrounded by K_2_CN^+^, as shown in Figure 2d. The highest intensities of K_4_PO_3_^+^ were found in alveolar edges, where phosphate-rich type-2 pneumocytes are quite often located. Thus, the K_4_PO_3_^+^ distribution resembled the distribution of phosphate-rich areas as revealed by IBM and most likely represents phospholipids (i.e., type-2 pneumocytes) or nucleic acids (i.e., nuclei).

The ZrO^+^ image (*m*/*z* = 105.86, Figure 2c) presents the distribution of the nanoparticles. The diameters of ZrO^+^ containing areas ranged from about 400 nm (corresponding to the size of a single pixel) up to 10 µm. As the primary particle size was 9 nm and the mean size of agglomerates within the instillation fluid amounted to 80 nm, the occurrence of more than one coherent ZrO^+^-positive pixel suggests the presence of accumulated nanoparticle agglomerates. These agglomerations might have formed in the absence of cells, i.e., secondary to the instillation process when instillation fluid is resorbed and particles contact the lung surfactant or lung lining fluid.

A correlation analysis overlaying the different components in different colors was performed (Figure 2d). The correlation analysis presents the tissue material marker ion K_2_CN^+^ in red, the ZrO^+^ signal in green, and the K_4_PO_3_^+^ in blue. K_4_PO_3_^+^ was predominantly found within the tissue at intense K_2_CN^+^ signals. This indicated a correlation with dense tissue and related most likely to compartments rich in nucleic acids or phospholipids. The ZrO_2_ nanoparticles were likely to have accumulated alongside alveolar walls or were taken up by macrophage-like cells (Figure 2d, white arrow) typical sizes [8]. Some smaller signals appeared to be in the alveolar spaces. It cannot be excluded that some nanoparticles may have been displaced during the sectioning process. However, most of these were still associated with tissue material of lower signal intensity.

### 2.3. Individual Position: Organic Depth Profiling

While the ToF-SIMS approach using O_2_^+^ sputtering results in excellent resolution and low detection thresholds, the extensive amount of fragmentation induced by the highly energetic O_2_^+^ primary ions on organic molecules is less ideal for the detection of organic molecules. In particular, large biomolecules are prone to fragmentation. However, studying the distribution of these molecules may contain valuable information on the effects of nanoparticles in tissues. Although it is expected to result in a higher detection limit, e.g., for Zr secondary ions, sputtering with large gas clusters leads to a much better yield for larger molecules and larger fragments [9,10]. In particular, Ar-Cluster sputtering leads to less fragmentation along with higher sputter yields per ion compared to smaller clusters. In the next chapter, Ar_1000_^+^ cluster sputtering is carried out and results will be compared to those of O_2_^+^ sputtering.

**Figure 3:**
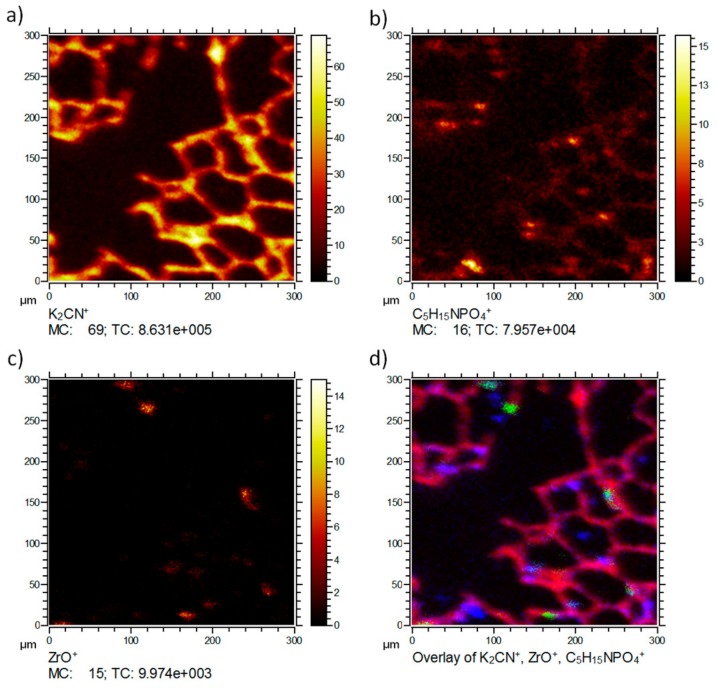
ToF-SIMS 3D analysis of an air-dried lung section three days post instillation of ZrO_2_ nanoparticles. Lateral distributions of three secondary ions (**a**) K_2_CN^+^; (**b**) C_5_H_15_NPO_4_^+^ (*m/z* = 184.09), and (**c**) ZrO^+^ is shown upon Ar-cluster sputtering. A correlation analysis of all ions is shown in (**d**), with K_2_CN^+^ in red, the C_5_H_15_NPO_4_^+^ in blue and the ZrO^+^ in green. The lateral distribution of K_2_CN^+^ shows the typical lung tissue structure. The phosphocholine fragment C_5_H_15_NPO_4_^+^ occurs in intense patches embedded in the septal structure (resembling the distribution of type-2 cells). The ZrO^+^ signals occurs in discrete intense areas sized 10–20 µm, which appear attached to the septal structure 10 µm. (MC: maximum counts per pixel, TC: total counts for the image).

Figure 3 shows the distribution of the typical signals of the same lung tissue section. Besides the tissue marker ion K_2_CN^+^ (a) and the NP-related ZrO^+^ (c), the distribution of the phosphatidylcholine head group C_5_H_15_NPO_4_^+^ (b) is shown as an example for larger organic molecular species, which are not extensively fragmented but detected intact. C_5_H_15_NPO_4_^+^ is a typical fragment of the important membrane lipid phosphatidylcholine. Consequently, the lateral distribution reveals the presence of this signal predominantly on the tissue material as expected for a membrane lipid. Several small patches of high intensity are visible. These most likely indicate the presence of pneumocyte type-2 cells, which produce the lung surfactant whose major phospholipid is phosphatidylcholine [11,12]. Of note, C_5_H_15_NPO_4_^+^ was not associated with accumulated ZrO^+^ (d), suggesting that (1) these NP, despite their large BET(Brunauer-Emmet-Teller) surface do not extensively bind lung surfactant, and (2) are not incorporated by type-2 pneumocytes.

**Figure 4:**
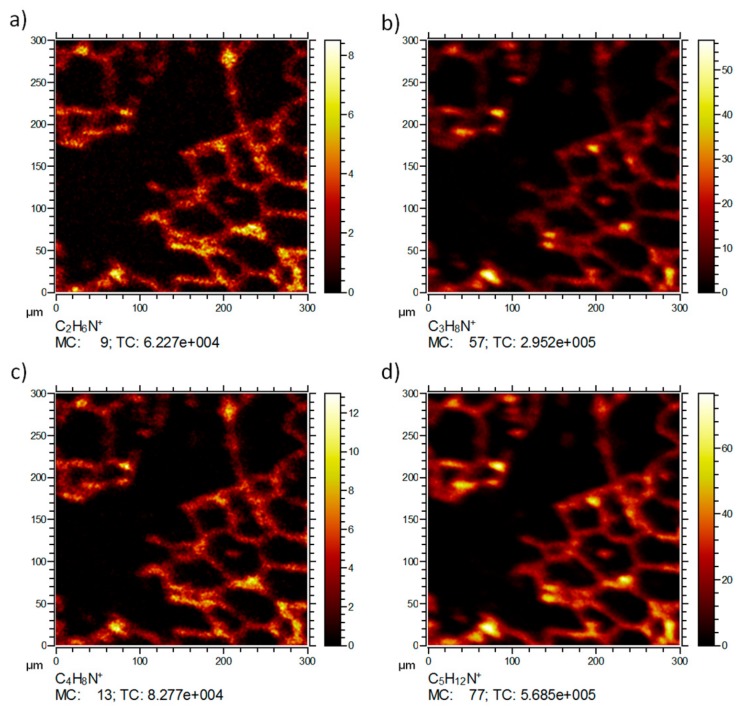
ToF-SIMS 3D analysis of an air-dried lung section three days post instillation of ZrO_2_ nanoparticles. Same site of analysis as shown in Figure 3. The lateral distribution of (**a**) C_2_H_6_N^+^; (**b**) C_3_H_8_N^+^; (**c**) C_4_H_8_N^+^; (**d**) C_6_H_12_N^+^ obtained by Ar-cluster sputtering is shown. (**a**) C_2_H_6_N^+^; (**b**) C_3_H_8_N^+^; fragments in (**a**,**c**) and most likely originate from amino acids; fragments in (**b**,**c**) originate from phosphatidylcholine fragments. MC: maximum counts per pixel, TC: total counts for the image.

Figure 4 shows the distributions of signals most likely related to fragments of amino acids or other tissue molecules. While no distinct localization patterns were obtained for C_2_H_6_N^+^ (*m*/*z* 44.05, Figure 4a), most likely due to the ubiquitous occurrence of amino acids throughout all cells, other fragments (C_3_H_8_N^+^ (*m*/*z* 58.06, Figure 4b), C_4_H_8_N^+^ (*m*/*z* 70.07, Figure 4c), C_5_H_12_N^+^ (*m*/*z* 86.10, Figure 4d)) show a pattern resembling that of C_5_H_15_NPO_4_^+^ from which they may have originated (at least in part). All these distributions reflect the septal structure of the lung tissue (compare K_2_CN^+^ in Figure 3a) and were not associated with ZrO_2_ NP.

### 2.4. Sequential Analysis of Identical Sample Sites: Ion Beam Microscopy (IBM)

To compare the results from IBM and ToF-SIMS analyses, a sequential analysis of the same site of ZrO_2_-NP laden lung tissue was carried out. Since the ToF-SIMS using Ar-cluster or O_2_ sputtering is a material-consuming (destructive) technique, IBM was done prior to ToF-SIMS analysis.

**Figure 5:**
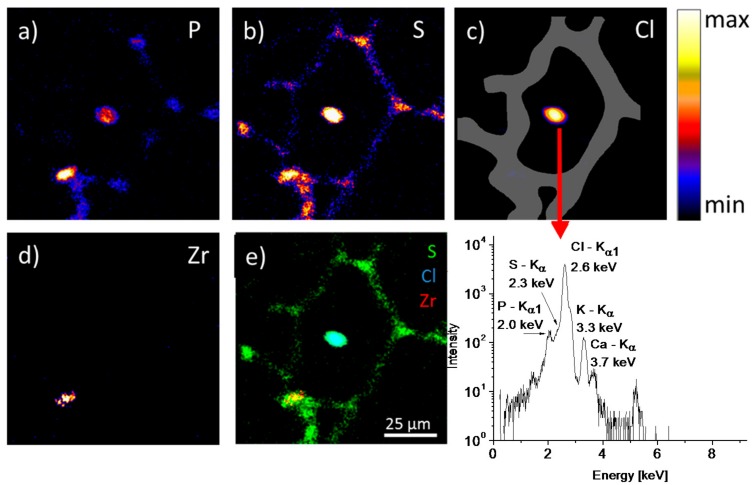
PIXE images of a ZrO_2_ laden lung tissue section 3 days post instillation. Images (**a**–**d**) show the elemental distribution patterns of the elements P, S, Cl and Zr in lung tissue (field-of-view: 100 × 100 µm²). The overlay image (**e**) reveals the co-localization of S with Cl and Zr elements. The intense Cl signal (**c**) in the center interferes with the signal of S. The interference is revealed by the PIXE emission spectrum from the central area (**f**).

The IBM results show a notable distribution of P and S signals within the tissue section (Figure 5a,b). Phosphorus reveals a patch pattern within the continuously distributed sulfur. Both elements can be used to visualize lung tissue. The origin of the phosphorus signals are most likely phospholipids from the tissue membranes or/and the phosphate of the nucleic acid backbone, which are most concentrated within the nuclei of the cells. Sulfur is a general marker for cells as it occurs in almost all proteins and cytoskeleton. The alveolar walls can be identified by the sulfur distribution indicating the localization of the pneumocytes, macrophages and other cells. An enhanced occurrence of sulfur and phosphorus elements was found in the alveolar junctions. The intense spots of the P and S distributions in the alveolar junction region could indicate the presence of pneumocyte type-2 cells, where their location is normally expected.

Surprisingly, the central area showed a highly intense chlorine signal co-localized with S and P signals (Figure 5c,e). As the S signal was strongly interfered with by the intense Cl emission, it was regarded as an artifact. The P signal, however, was partially separated from the Cl signal (as can be seen from the emission spectrum (Figure 5f)) and indicated the validity of the assignment in the P image. Furthermore, some Ca and K signals were found at that position, which suggested the presence of a salt residue. Of note, no corresponding structure was detected by ToF-SIMS.

The zirconium distribution in Figure 5d only has a single area of notable intensity. This area is found within the tissue structure. This intense signal is found co-localized with the sulfur intensity. The higher sulfur as well as the phosphorous content compared with that in surrounding tissue could be usually found in macrophages. Furthermore the spatially defined appearance of the zirconium signal in a single area with a diameter of about 10 µm suggests also that the ZrO_2_ nanoparticles were accumulated and might be contained in a macrophage. The achieved lateral resolution of about 1 µm revealed the substructure of Zr agglomerates inside a cell.

### 2.5. Sequential Analysis of Identical Sample Sites: ToF-SIMS

**Figure 6:**
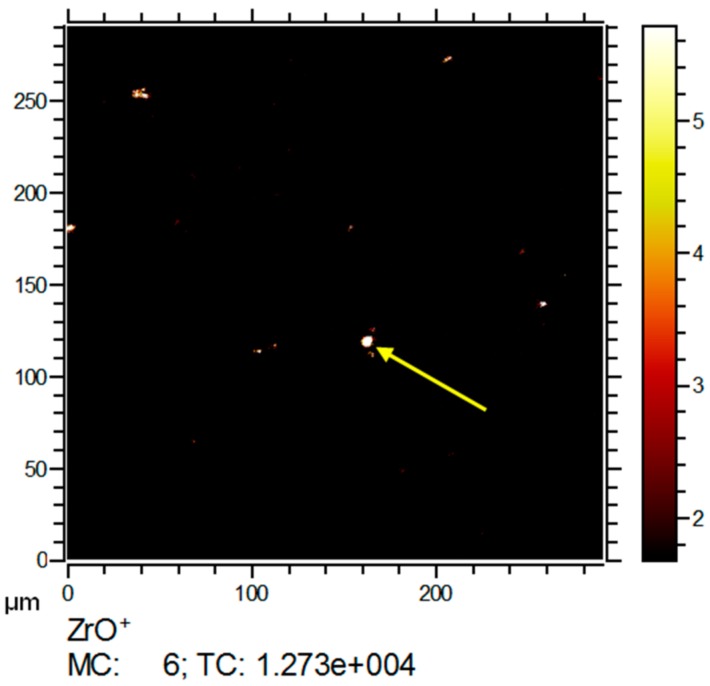
ToF-SIMS 3D analysis of the ZrO_2_ NP containing lung section previously subjected to IBM measurement (see Figure 5). The lateral distribution of ZrO^+^ reveals several intense signals; image contrast was enhanced to visualize small signals. The central ZrO^+^ signal (yellow arrow) corresponds to the Zr signal in Figure 5. MC: maximum counts per pixel, TC: total counts for the image.

**Figure 7:**
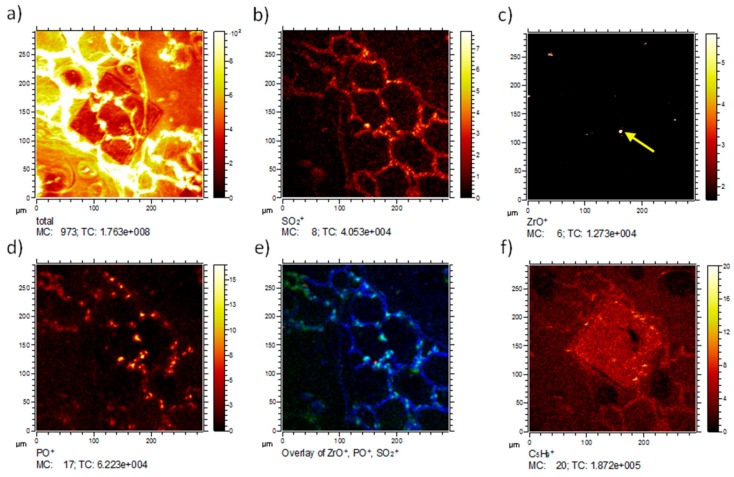
ToF-SIMS 3D analysis of a ZrO_2_ NP containing lung section three days post-instillation, previously subjected to IBM measurement (see Figure 5). Lateral distributions of selected secondary ions. (**a**) The total ion distribution clearly indicates the area of proton bombardment during IBM analysis; (**b**) The SO_2_^+^ distribution resembles the tissue structure; (**c**) The lateral distribution of ^90^ZrO^+^ reveals several intense signals. The central Zr signal (yellow arrow) corresponds to the Zr signal in Figure 5; (**d**) The PO^+^ image indicates the presence of phosphorus related compounds, which are typically nuclei and membrane lipids; (**e**) The red-green-blue (RGB) overlay shows the localization of the ZrO^+^ (red), PO^+^ (green) and SO_2_^+^ (blue); (**f**) The C_5_H_9_ distribution reveals elevated levels of small organic molecules indicating fragmentation of larger molecules in the area that was treated with the ion beam. (MC: maximum counts per pixel, TC: total counts for the image).

**Figure 8:**
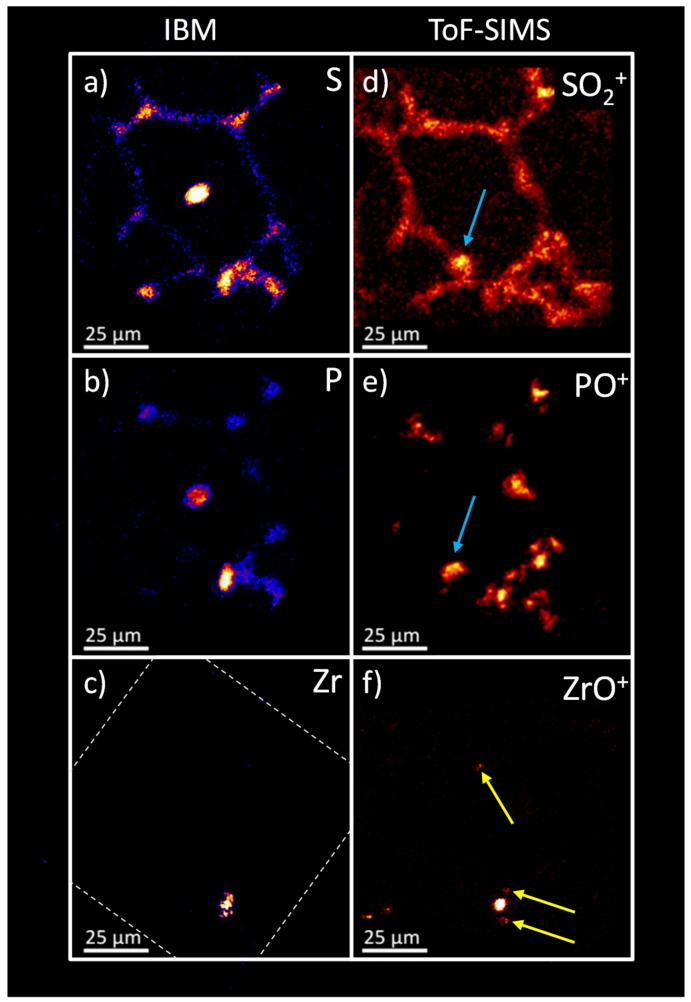
Comparison of the IBM and ToF-SIMS lateral distributions for selected signals side-by-side in the ZrO_2_ NP containing lung section of Figure 5. The IBM distributions (**a**–**c**) were tilted to enable a direct comparison to the ToF-SIMS images (**d**–**f**, magnified). All distributions show a reasonably high congruency. The central, intense area of the S and P distribution from the IBM results has no direct equivalent in the ToF-SIMS results (interpreted as an artifact, see text). However, an area of similar size and shape is found in the lower left of the alveolar structure in the SO_2_^+^ and PO^+^ ToF-SIMS images (blue arrows). The border of the field-of-view of the IBM is indicated by a dashed line in (**c**).

Results of the sequential analyses carried out first by IBM and than by ToF-SIMS are shown in Figure 6, Figure 7 and Figure 8. Treatment with the high-energy proton beam led to locally confined changes of the tissue surface (Figure 7a,f), which were used to identify the region-of-interest for the subsequent ToF-SIMS measurement. To maximize the signal yields especially for zirconium, we chose the more fragmenting O_2_^+^ ion beam sputter method for the comparative analysis, as it increases the yields for electropositive elements.

Furthermore, the lateral distributions of the signals from zirconium derived ions (ZrO_x_^+^) were analyzed. The respective isotopic signal distributions were used to ensure the identity of the Zr derived signals combined (compare Appendix A). Figure 6 shows several intense Zr signals with diameters below 1 µm and up to 9 µm. As the pixel size limits the minimum diameter for signals to about 600 nm, smaller nanoparticles appeared as single pixels.

The lateral distribution of the total ion intensity reveals the overall structure of the analyzed tissue (Figure 7a). The area subjected to IBM analysis was easily recognized due to its reduced total secondary ion yield and the increased levels of small organic fragments (Figure 7f). To better compare ToF-SIMS and IBM images, we analyzed the distribution of S and P using SO_2_^+^ (*m*/*z* 63.97, Figure 7b) and PO^+^ (*m*/*z* 46.96, Figure 7d) signals, which show lung tissue typical distributions if compared to the analyses shown above. A comprehensive image is presented as a RGB overlay, and shows the tissue-related SO_2_^+^ signal in blue, the phosphate signal in green, and the Zr oxide particle signal in red (Figure 7e). The RGB image shows agglomerated ZrO_2_ NP in the tissue, revealing that most nanoparticles were directly associated with the tissue.

As the ion-beam-treated area was visible on ToF-SIMS images, we speculated that the sample was altered during the IBM analysis. A careful evaluation of ToF-SIMS analyses from ion-beam-treated sites showed that a slightly higher amount of small organic fragments (e.g., C_5_H_9_ (*m*/*z* 69.07, Figure 7f)) were detected in the ion beam-treated area and its close vicinity. It is conceivable that due to the impact of high energy protons, alterations of the organic material occurred. This would especially be the case with breaking of chemical bonds (similar to the proton beam writing process) [13,14], as it would potentially explain the slightly elevated levels of small organic fragments. This shows that the IBM technique could at least gradually change the molecular composition of tissue components, and this aspect is of importance for the direct comparison of results.

### 2.6. Sequential Analysis of Identical Sample Sites: Comparison of IBM vs. ToF-SIMS 

The results of the IBM analysis were compared to ToF-SIMS results (Figure 8). Elemental distributions of the X-ray emission on the left (a–c) were compared to the oxygen containing secondary ion distributions on the right (d–f). For S and SO_2_^+^, a high degree of correlation was obvious. With both techniques, a relatively weak signal distribution resembled the tissue structure with several higher intensity signals at the alveolar junctions. However, a difference was seen in the intense signal in the centre of the PIXE image (Figure 8a,b). This central structure consisted of K, Ca, P, and Cl that formed a salt deposit as a result of the preparation process, and appeared to be an artifact (see above). Also, an indication for this deposit was found neither on micrographs taken after the IBM analysis (not shown), nor in the ToF-SIMS analysis. As the structure was found in the centre of an alveolus, where it was not linked to the tissue material, we assumed that this salt deposit was moved during transport. However, the ToF-SIMS distributions of SO_2_^+^ and PO^+^ both revealed an area similar in size and shape in the lower left corner of the alveolar structure (indicated by blue arrows) which might represent the salt deposit at its new position.

The P and PO^+^ distribution overall was highly similar. However, the ToF-SIMS image apparently presented the intense signals of the IBM at a higher resolution and enabled a more detailed assessment of the substructure compared to the IBM.

With respect to the Zr/ZrO^+^ signals, a coincidence was obtained for the highly intense signal spot seen in the lower parts of Figure 8c,f, although the detailed structure of Zr differed for both methods. Interestingly, the ToF-SIMS analysis revealed additional small ZrO^+^ signals (Figure 8f, yellow arrows), suggesting a lower limit of detection for the ToF-SIMS analysis along with a better lateral resolution. In fact, the routinely achieved lateral resolution in IBM experiments is about 1 µm, with limits of detection being in the parts-per-million (ppm) range [15,16]. In contrast, a lateral resolution of less than 30 nm (under ideal conditions) and limits of detection in the ppm-ppb (parts-per-billion) range may be achieved with ToF-SIMS analysis [17,18]. However, for both techniques the analytical parameters might still show some room for improvements. Prolonged measurement times and high performance detectors might lead to higher signal intensities and lower limits of detection in both instruments, but these were not available for this study. The application of high performance PIXE detectors will result in improved limits of detection and reduce measurement times. For ToF-SIMS, the amount of material eroded by the sputter beam per analysis cycle could be reduced to enhance the amount of material exposed to the analysis beam. It can be assumed that in particular for this analysis more signal intensity could be revealed under extended measurement conditions, since the majority of the signal intensity of the nanoparticles is only acquired towards the end of the analysis. Also, the lateral resolution (limited by the pixel number at a given Field-of-view to achieve a reasonably fast analysis) could be significantly improved for the detection of smaller agglomerates by selecting a smaller field-of-view or raising the pixel number per length unit. However, in favor of analyzing a larger area and measurement time constraints, a lateral resolution similar to the IBM was selected for this study.

The IBM provides element (inorganic) information [2,19,20,21], whereas ToF-SIMS detects inorganic and organic species [22]. The detection of molecular information is only possible by ToF-SIMS, and works especially well for Ar-cluster ions. Therefore, in contrast to the IBM, ToF-SIMS also can serve for speciation analyses [23,24]. However, the certainty for the identification for even smaller quantities of small nanomaterials (i.e., weak signals) in a complex matrix in ToF-SIMS is challenging, because interfering signals could hinder the separation of small partially overlapping NP-related signals in crowded mass spectra. In IBM, the certainty of the identification depends on the assignment and selection of the correct emission lines and is comparatively high.

Quantitative information is routinely obtained for the IBM measurements without the need for standards or prior knowledge on the sample composition. In ToF-SIMS, quantitative information is only achievable by great efforts for simple, highly characterized samples (e.g., silicon wafers doped with boron) with the help of matrix-matched reference samples. For complex tissue samples, no simple routines exist for the quantification only based on ToF-SIMS results. Matrix effects are reported for ToF-SIMS as well, whereas the IBM does not suffer from severe matrix effects.

Depth information in IBM analyses is directly obtained via the RBS spectrometry, which also provides direct information if materials are internalized into cells or covered by tissue material. Depth resolutions of 100 nm are achieved [2,19]. In ToF-SIMS, depth information is accessed via repeated cycles of surface analysis and surface erosion. Consequently, information on the internalization of materials can be obtained by interpreting the appearance of signals in successive scans. Furthermore, depth distributions as well as 3D distributions of signals can be analyzed. Depth resolutions of about 20 and 10 nm were published for low energy Cs and Ar-cluster ion sputtering of multilayer amino acid films, respectively [25]. However, this excellent resolution comes at the cost of analysis time, which is increasing with lower energy ions (i.e., lower sputter yields).

Both ToF-SIMS and ion beam microscopy need vacuum conditions. Vacuum compatibility for biological samples can be achieved by chemical fixation or analysis in the frozen state [26,27,28].

The influence of the proton beam on the samples integrity was estimated as the site of the analysis is visible in the successive ToF-SIMS images and a slightly higher amount of small organic fragments was found in the irradiated area. However, the extent of the damaging influence was not fully assessed during this study (compare Figure 7f). In comparison, ToF-SIMS 3D imaging, by nature, is a sample consuming technique. This is because signals can only be acquired if the respective materials are sputtered and ionized. Especially for the sensitive detection of low amounts of particle within complex matrices, it is necessary to obtain as much intensity as possible i.e., consume as much material as possible to ensure a comprehensive detection of all nanoparticles.

While ToF-SIMS already is a large and complex instrument, the IBM is still much more demanding in terms of size and complexity. Consequently, the pricing of commercially available ToF-SIMS instruments is much lower than for the IBM instruments, which need elaborate particle accelerators and a variety of detection components and are not available as complete kits. The time requirements for ToF-SIMS analyses of a single biological tissue area under the conditions shown here typically are in the order of 8–16 h (depending on the depth resolution and sample thickness). For the IBM measurements, approximately one hour is needed to reach ppm detection limits, however, these large-scale devices usually are shared among several institutions. In consequence, “beam time” for measurements is precious.

In summary, each technique revealed the position of nanoparticle agglomerations within a tissue section individually and in a successive analysis of the same section. Significant congruency in the lateral distributions for phosphorous, sulfur and zirconium enabled a cross-validation of the results of both techniques. Due to its non-consuming nature and high lateral resolution, the IBM analysis enhances the confidence for the assignments of low intensity signals (especially for nanomaterials) in ToF-SIMS analyses and allows a cross-validation for early ToF-SIMS method development studies. 

A promising strategy for achieving quantitative information in ToF-SIMS might be achieved by using IBM and ToF-SIMS for selected samples to obtain quantitative information and establish sensitivity factors for defined experimental conditions/sample matrices.

## 3. Conclusions

The detection of ZrO_2_ nanoparticles in a real-world sample (lung tissue section from a toxicity study) was successfully shown by both ion beam techniques, IBM and ToF-SIMS. It is of toxicological relevance that with both techniques, ZrO_2_ NP occurred in the lung as agglomerates, most probably in phagocytic cells, whereas no or negligible small quantities were associated with the lung epithelium. Each technique revealed the position of nanoparticle agglomerations within a tissue section individually. ToF-SIMS additionally could reveal the distribution of molecular fragment signals, which is in general not possible by IBM. Significant congruency in the lateral distributions was found for the localization of phosphorous-, sulfur-, and zirconium-containing compounds for both techniques enabling a cross-validation of the results. A comparison of the results revealed similar lateral resolutions with a slight advantage on the ToF-SIMS side. Lower limits of detection for the ToF-SIMS analysis were found in this study, whereas for the IBM measurement quantitative information is obtained without sample consumption. In consequence, a successive analysis by both techniques delivers an extremely high confidence on the validity of the distributions of nanomaterials and other components in tissue sections at high lateral resolutions, along with quantitative information.

## 4. Materials and Methods 

### 4.1. ZrO_2_ Nanoparticles

ZrO_2_ nanoparticles investigated in this study had a primary size of 9–10 nm according to electron microscopy, a surface area of 117 m^2^/g, and were surface-coated with either tetraoxadecanoic acid or acrylate. A detailed characterization has been published previously [5].

### 4.2. Animal Experiments and Lung Tissue Preparation

Preparation of NP suspensions for instillation and instillation experiments were described before [6]. In brief, tetraoxadecanoic acid or acrylate-coated ZrO_2_ NP were suspended in sterile H_2_O, coated with rat serum albumin to prevent agglomeration, and further diluted in the instillation fluid (25 mM sodium bicarbonate gassed with 5% CO_2_ to pH 7.4–7.8); 0.5 mL of this fluid containing 1.2 or 2.4 mg of either ZrO_2_ NP quality was intratracheally instilled under isoflurane anaesthesia. Particle size in the instillation fluid as measured by optical tracking methods was 71–80 nm [6]. Animals used in this study were deeply anaesthetized with ketamine and xylazine, and bled via the Aorta descendens three days post-instillation. The left lung was inflated with 3 mL Cryomatrix (Thermo Shandon Ltd., Runcorn, UK), snap frozen in liquid nitrogen, and stored at −80 °C. Seven µm-thick cryo-sections were cut from the hilar region of the left lung. Sections investigated by ToF-SIMS were from rat lungs laden with 2.4 mg acrylate-coated ZrO_2_ (see Figure 2, Figure 3 and Figure 4), were mounted onto room temperature indium tin oxide-coated slides. After drying, sections were kept frozen and transferred to the pre-cooled chamber of the ToF-SIMS instrument, where they were dried completely under vacuum (pressure < 1 × 10^−6^ mbar). Sections used for IBM or for the comparison of IBM and ToF-SIMS were from rat lungs laden with 1.2 mg tetraoxadecanoic acid-coated ZrO_2_ (see Figure 1 and Figure 5, Figure 6, Figure 7 and Figure 8). Sections were mounted onto a room temperature polypropylene foil fixed in custom made metal frames fitting into the IBM sample chamber. After drying, sections were transported on dry ice, immersed in cold methanol for 10 min, and dried prior to IBM investigation.

### 4.3. ToF-SIMS Analysis—Sputter Conditions

Measurements using O_2_ sputtering were performed at a TOF-SIMS^5^ (ION-TOF, Münster, Germany). An ion dose of Bi_3_ at 25 keV of about 1.3 × 10^11^ ions was applied to an analysis raster of 200 × 200 µm² or rather 300 × 300 µm² with a pixel raster of 512 × 512 pixels. O_2_ was used as a sputter ion at an energy of 1 keV. A total sputter dose of 2.2–4.7 × 10^15^ ions was applied to a sputter raster size of 600 × 600 µm² or rather 900 × 900 µm². The delayed extraction mode in combination with non-interlaced sputtering at a cycle time of 70 µs resulting in a mass range up to 350 Da was used. The calibration of the mass spectra was facilitated by the use of Na^+^, K^+^, Na_2_CN^+^, Na_2_CNO^+^, and K_2_OH^+^. Mass resolutions (*R = m*/Δ*m*) between 2000 and 7000 were achieved.

Measurements using Ar-cluster sputtering were performed at an upgraded TOF-SIMS^4^ instrument (ION-TOF, Münster, Germany). An ion dose of Bi_3_ at 25 keV of 1.67 × 10^9^ ions was applied to an analysis raster of 300 × 300 µm^2^ with a pixel raster of 256 × 256 pixels. Ar_1000_ clusters were used as sputter ions at an energy of 10 keV. A total sputter dose of 1.13 × 10^13^ ions was applied to a sputter raster size of 500 × 500 µm^2^. The non-interlaced sputtering mode was used at a cycle time of 200 µs resulting in a mass range up to 1500 Da was used. The calibration of the spectra was facilitated by the use of the small organic ions (CH_3_^+^, C_2_H_5_^+^, C_3_H_7_^+^, and C_4_H_9_^+^). Mass resolutions (*R = m*/Δ*m*) between 3100 and 9000 were achieved.

The resulting 3D distributions of the signals were projected onto a 2D plane to enable a simple comparison of the signals.

### 4.4. Ion Beam Microscope Analysis

Ion beam measurements were conducted at the Leipzig Ion Nanoprobe LIPSION (Leipzig, Saxony, Germany) of the Felix-Bloch-Institute for Solid State Physics. The measurements were executed with a proton beam of an energy of 2.25 MeV with a beam current of about 1 nA under vacuum conditions of 5.0 × 10^−3^–1.0 × 10^−5^ Pa. The spatial resolution was set to approximately 1 µm.

The PIXE spectroscopy employed a Canberra PIXE detector (Meriden, CT, USA) consisting of a high purity Germanium crystal (95 mm^2^ active area) additionally covered with a 60 μm polyethylene layer, in order to avoid the penetration of backscattered protons.

For the RBS spectrometry measurements, the backscattered protons were detected using a passivated implanted planar silicon (PIPS)-detector from Canberra (Meriden, CT, USA). Rutherford backscattering spectrometry was applied to determine accumulated charge, atom density (atoms/cm^2^) and element matrix composition (C, N, and O). These data were used as input parameters to calculate the concentration of ZrO_2_ in ng/cm² from the integral intensity of the Zr K-line from the PIXE spectrum [2,16,29].

The data was collected using the MpSys Software (MARC Group of Prof Jamieson, Melbourne, Australia). The charge, density and element concentration were determined by fitting the experimental RBS and PIXE data in SIMNRA 6.0 (Mayer, M., Garching, Germany) and GeoPIXE5.1b software (CSIRSO, Melbourne, Australia).

## Figures and Tables

**Figure A1:**
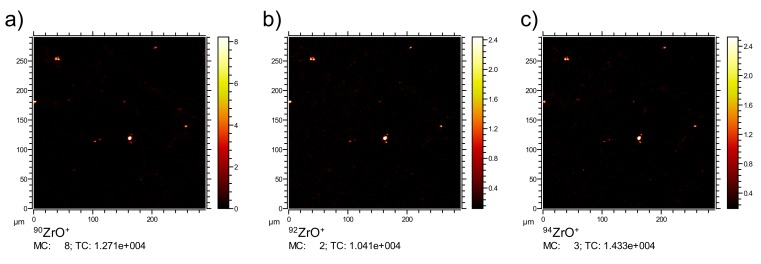
Correlating signal distributions of ZrO_2_-related isotopes. The lateral signal distributions of the main ZrO isotopes ^90^ZrO^+^ (**a**) ^92^ZrO^+^ (**b**) and ^94^ZrO^+^ (**c**) are in agreement with each other. Consequently, all the observed signals are derived from the same ZrO_2_ source and the identity of the ZrO_2_ particles is confirmed.
